# Chimeric antigen receptor (CAR)-modified natural killer cell-based immunotherapy and immunological synapse formation in cancer and HIV

**DOI:** 10.1007/s13238-017-0415-5

**Published:** 2017-05-09

**Authors:** Dongfang Liu, Shuo Tian, Kai Zhang, Wei Xiong, Ndongala Michel Lubaki, Zhiying Chen, Weidong Han

**Affiliations:** 1 Center for Inflammation and Epigenetics, Houston Methodist Research Institute, Houston, TX 77030 USA; 2 Department of Microbiology and Immunology, Weill Cornell Medical College, Cornell University, New York, NY 10065 USA; 3 Institute of Basic Medicine, College of Life Sciences, Chinese PLA General Hospital, Beijing, 100853 China

**Keywords:** natural killer cell, chimeric antigen receptor, immunotherapy, immunological synapse, HIV, cancer

## Abstract

Cytotoxic T lymphocytes (CTLs) and natural killer (NK) cells contribute to the body’s immune defenses. Current chimeric antigen receptor (CAR)-modified T cell immunotherapy shows strong promise for treating various cancers and infectious diseases. Although CAR-modified NK cell immunotherapy is rapidly gaining attention, its clinical applications are mainly focused on preclinical investigations using the NK92 cell line. Despite recent advances in CAR-modified T cell immunotherapy, cost and severe toxicity have hindered its widespread use. To alleviate these disadvantages of CAR-modified T cell immunotherapy, additional cytotoxic cell-mediated immunotherapies are urgently needed. The unique biology of NK cells allows them to serve as a safe, effective, alternative immunotherapeutic strategy to CAR-modified T cells in the clinic. While the fundamental mechanisms underlying the cytotoxicity and side effects of CAR-modified T and NK cell immunotherapies remain poorly understood, the formation of the immunological synapse (IS) between CAR-modified T or NK cells and their susceptible target cells is known to be essential. The role of the IS in CAR T and NK cell immunotherapies will allow scientists to harness the power of CAR-modified T and NK cells to treat cancer and infectious diseases. In this review, we highlight the potential applications of CAR-modified NK cells to treat cancer and human immunodeficiency virus (HIV), and discuss the challenges and possible future directions of CAR-modified NK cell immunotherapy, as well as the importance of understanding the molecular mechanisms of CAR-modified T cell- or NK cell-mediated cytotoxicity and side effects, with a focus on the CAR-modified NK cell IS.

## INTRODUCTION

Natural killer (NK) cells were discovered in the 1970s (Herberman et al., 1975a, b; Kiessling et al., 1975a, b) but are not considered a main research area in the field of immunology (Yokoyama, 2008). For decades, NK cells have lived in the shadow of T cells and other immune cells. In the early days of NK cell discovery, many immunologists did not appreciate the importance of NK cells to the body’s defense system. However, the essential roles of NK cells in the immune system are revealed in patients with NK deficiency (Orange, 2013), who have increased rates of malignancy (Orange, 2013; Morvan and Lanier, 2016) and are susceptible to herpesvirus infections, including varicella pneumonia, disseminated cytomegalovirus, and herpes simplex virus (Biron et al., 1989). Similar results were described in mice with impaired NK activity (Talmadge et al., 1980; Morvan and Lanier, 2016), which highlights the importance of NK cells in the immune system.

Chimeric antigen receptor (CAR)-modified T cell therapy has become a promising immunotherapeutic strategy for the treatment of blood cancers (Porter et al., 2011; Kim et al., 2016; Maude and Barrett, 2016) and has gained the significant attention of researchers in both academia and industry (Glienke et al., 2015). In 1989, Gross and colleagues described the concept of expressing antibody in a cytotoxic T cell hybridoma (Gross et al., 1989). Progressive advances in construction of chimeric T cell receptors, including introduction of a single costimulatory molecule, in the past 25-year development by several groups on the basis of this concept have led to rapid progress on CAR Therapy. For example, anti-CD19 CAR T cell therapy was granted “breakthrough therapy” by the United States Food and Drug Administration (FDA) in 2014 (Gill and June, 2015; Gill et al., 2016).

NK cells were originally described for their capacity to spontaneously kill tumor cells (Rosenberg et al., 1974; Herberman et al., 1975a, b; Kiessling et al., 1975a, b), which differ from T cells that require prior sensitization. As their name implies, NK cells kill susceptible target cells, such as virus-infected cells or tumor cells, without prior sensitization. Unlike T cells, the activation of NK cells is controlled by the integration of both stimulatory and inhibitory receptors (Bakker et al., 2000; Long et al., 2013). Similar to CAR-T technology, CAR-NK cell strategy involves isolating a patient’s own NK cells or expanding commercially available NK cell lines (e.g., NK92 cells), engineering these cells in a good manufacturing practice (GMP) laboratory to express CAR, which recognize a tumor-specific protein, and re-infusing the engineered NK cells back into the patient.

The cell biology and immunology of NK cells have been extensively discussed in other reviews (Lanier, 2005; Vivier et al., 2011; Lam and Lanier, 2016). This review focuses on CAR-modified NK cell immunotherapy and the CAR-modified NK immunological synapse (IS). This review is divided into the following five main sections: (1) the rationale for the development of CAR-modified NK cell-based immunotherapy; (2) CAR-modified NK cell-based immunotherapy to treat cancer; (3) CAR-modified NK cell-based immunotherapy to treat infectious diseases; (4) the CAR-modified NK cell IS; and (5) perspectives. The main messages that this review conveys are the following:

1. CAR-modified NK cells are a promising intervention for the treatment of cancer and infectious diseases.
2. The CAR-modified NK cell IS is important to the understanding of CAR-modified NK cell-mediated cytotoxicity and side effects.

## THE RATIONALE FOR THE DEVELOPMENT OF CAR-MODIFIED NK CELL-BASED IMMUNOTHERAPY

In addition to T cell-mediated immunotherapy, the unique biology of NK cells makes them a valid tool for immunotherapy (Morvan and Lanier, 2016). Clinically, NK cells from peripheral blood can be defined as CD3-negative and CD56-positive peripheral blood mononuclear cells (PBMCs). There are several advantages of using NK cells as an immunotherapeutic strategy to treat cancer and infectious diseases, as described below.

### NK cell activation does not require prior sensitization or human leukocyte antigen matching

It is well known that NK cell activation is controlled by the integration of stimulatory and inhibitory signals (Long et al., 2013). CTL activation requires a sensitization phase, in which unprimed T cells interact with antigen-presenting cells (APCs) to become activated lymphocytes. These activated CTLs then enter the effector phase in which they eliminate virus-infected target cells or tumor cells. However, NK cells can kill virus-infected target cells and tumor cells without prior sensitization. Specifically, if the signaling of an activating receptor, such as CD16 or natural killer group 2, member D (NKG2D), dominates, NK cells will kill target cells without prior sensitization, which provide the rapid, first-line defense mechanism. This feature of NK cell biology makes these cells an effective and valid tool for rapidly eliminating virus-infected cells or tumor cells, serving as the body’s first line of defense. Another feature of CTL-mediated killing is “major histocompatibility complex (MHC) restriction”, in which the T cell receptor (TCR) must recognize its self-MHC molecule that is expressed on antigen-specific target cells, such as virus-infected cells (Ada, 1994). However, NK cell-mediated cytotoxicity is “MHC unrestricted”, which means that NK cells kill target cells that don’t express MHC class I molecules, a guiding principle in the NK field, also known as the “missing self” hypothesis (Karre et al., 1986; Ljunggren and Karre, 1990). NK cell activation is controlled by the strength of the stimulatory receptor signal. The principle of CAR immune cell design matches the mechanism of NK cell activation. CAR-modified NK cells can provide strong activating signals by linking the antigen-specific single-chain variable fragment (scFv) domain with CD3zeta, an essential intracellular signaling molecule for NK cell activation (Lanier, 2005; Watzl and Long, 2010). Given the unique NK cell mechanism for killing target cells, CAR-modified NK cells provide attractive effector cells for immunotherapy.

### NK cell killing does not require antigen-specific receptors, such as TCRs for CTLs

As described above, NK cell cytotoxicity is triggered by various germline-encoded stimulatory receptors (Vivier et al., 2008) and does not require the highly polymorphic TCR. A potential concern of genetically modified T cells with tumor- or virus-specific TCRs is “TCR mispairing” (Kershaw et al., 2005), which occurs when introduced alpha and beta TCR chains mispair with endogenous TCR chains. This process not only creates mismatched heterodimers of unknown specificity, but also reduces the cell-surface density of tumor- or virus-reactive TCRs (Aggen et al., 2012). To avoid “TCR mispairing”, Aggen and colleagues developed single-chain T cell receptor variable fragment (scTv), which links the variable domains of the alpha and beta TCR chains by a flexible linker, to generate high-affinity, stable TCR for MHC complexes associated with cancer and HIV (Aggen et al., 2012). Therefore, it is possible to modify NK cells with an antigen-specific scTv gene without concerns of “TCR mispairing”, a phenomenon commonly found in TCR-modified CTLs (Kershaw et al., 2005). Therefore, genetically modified NK cells expressing scTv are easier and more feasible to generate than T cells expressing exogenous TCRs.

### NK cell immunotherapy has less severe side effects

In contrast to T cells, CAR-modified NK cells show less severe side effects, such as graft-versus-host disease (GvHD), because donor NK cells usually do not attack non-hematopoietic tissues such as liver, kidney, muscle, and lung. A number of clinical trials showed that NK cell infusion has less severe GvHD than does T cell infusion. This clinical observation could also be related to the unique cell biology and immunology features of NK cells. For example, compared to T cells, conventional NK cells have a shorter life span, which can mitigate the risk of GvHD development in leukemia patients treated with NK cells (Miller et al., 2005; Curti et al., 2011). In addition to a shorter life span, NK cell expansion is tightly controlled by constitutively expressed inhibitory receptors, such as killer immunoglobulin-like receptor (KIR), CD94/ natural killer group 2A (NKG2A), and other inhibitory receptors (Long, 2008). These features of NK cells may explain why NK cells have less severe GvHD during NK cell infusion, compared with T cell infusion. However, a direct comparison between CAR-modified NK cells and CAR-modified T cells has not been performed *in vivo*.

### CAR-modified NK cells can be potentially used as an “off-the-shelf” universal CAR product

A pressing issue in the field of immunotherapy is whether an “off-the-shelf” universal CAR product can be developed. CAR-modified T cell products from individuals are costly and time consuming to prepare. Generation of “off-the-shelf” CAR-modified T cell products will significantly reduce the cost of immunotherapy. Using the clustered regularly interspaced short palindromic repeats (CRISPR) and CRISPR-associated protein 9 technique (Cong et al., 2013; Cong and Zhang, 2015), as well as other gene-editing technologies, to knockout endogenous TCRs and human leukocyte antigen (HLA) class I molecules for universal CAR-modified T cell generation is still in the preclinical phase of investigation (Ren et al., 2016; Liu et al., 2017b). Most of these strategies are in the concept phase. However, CAR-modified NK cell lines such as NK92 may lead to the development of feasible, “off-the-shelf” CAR products in the near future.

### Sufficient numbers of NK cells can be harvested from peripheral blood

A critical aspect of successful immunotherapy is to rapidly generate sufficient numbers of CAR-modified cells *in vitro*, which requires at least one million cells for expansion *ex vivo*. In general, there are sufficient numbers of NK cells that can be directly isolated from peripheral human blood. Around 10%–15% of PBMCs in the buffy coat layer are CD3-negative and CD56-positive NK cells. We usually can isolate between 10–20 million NK cells per buffy coat layer in each healthy individual. Immobilized apheresis products contain 5%–15% NK cells. To isolate NK cells, CD3^+^ cell depletion of PBMCs is commonly performed, followed by CD56^+^ cell enrichment using immunomagnetic bead separation with medical devices and clinical-grade reagents; these methods are feasibly conducted in the clinic due to the sufficient numbers of NK cells that can be isolated directly from peripheral blood.

### Functional NK cell lines are available

Compared to CAR-modified T cell-mediated immunotherapy, an advantage of NK cell-based immunotherapy is that functional, immortal NK cell lines are available. There are a number of functional NK cell lines that are cytotoxic and produce cytokines, such as NK92 (Gong et al., 1994), NKL (Robertson et al., 1996), KHYG-1 (Yagita et al., 2000), and YTS (Cohen et al., 1999; Klingemann et al., 2016). Among these NK cell lines, NK92 is the most promising cell line for clinical applications. NK92-mediated immunotherapy is now undergoing phase I/II clinical trials (Arai et al., 2008; Tonn et al., 2013). Commonly, NK92 cells must be irradiated prior to infusion to prevent permanent engraftment. The amount of irradiation required is around 10 Gy. The dose of irradiated NK92 infusion can be up to 10^10^ NK92 cells/m^2^. Importantly, irradiated NK92 cells have been proven safe for infusion in patients, as demonstrated by several NK92 clinical trials (NCT00900809, NCT00990717, NCT00995137, and NCT01974479). In contrast to CAR-modified T-mediated immunotherapy, there are few functional CTL cell lines available that can be used in clinical trials. Antigen-specific CTL clones can be expanded *ex vivo* for 2–3 months. After CTL clone expansion *in vitro*, many features of the CTL clones are altered, limiting their clinical application. In addition, there are few reports of the clinical applications of CTL clones to treat cancer and infectious diseases. Although several advantages of NK cells described above regarding NK-based immunotherapy, abnormal NK cell number and cytolytic functions have been proposed in various cancers (Costello et al., 2002; Farnault et al., 2012). Using functional CAR-modified NK cell lines is an important, alternative strategy to treat cancer and infectious diseases. In conclusion, to alleviate some disadvantages of CAR-modified T cell immunotherapy, additional NK-based CAR immunotherapy can be an alternative and effective approach for some patients. However, *in vivo* direct comparison between CAR-T and CAR-NK on the cytotoxicity, manufacture speed, proliferation capability, persistence, side effects etc., is urgently needed.

## CAR-MODIFIED NK CELL-BASED IMMUNOTHERAPY TO TREAT CANCER

Cancer is the leading cause of death worldwide: An estimated 8.2 million people die each year from cancer. A major public health problem in the United States, cancer is the second-leading cause of death (Siegel et al., 2016). In 2016, 1,685,210 new cancer cases and 595,690 cancer deaths were projected to occur in the United States (DeSantis et al., 2016; Siegel et al., 2016; Torre et al., 2016).

The clinical investigation of CAR-modified NK cell-based immunotherapy has been intensively conducted for several types of cancer (Rezvani and Rouce, 2015). Similar to CAR-T cell based immunotherapy, genetically modified NK cells using various CAR molecules to redirect different antigen specificity has been discussed by other reviews (Glienke et al., 2015; Hermanson and Kaufman, 2015; Rezvani and Rouce, 2015). This section will focus on the use of the CAR-modified NK92 cell line. Currently, CAR-modified NK92 cell line is used as effector cells for various cancer treatments, as detailed below:

### CAR-modified NK cells to treat acute lymphoblastic leukemia

CD5 is highly expressed in T cell acute lymphoblastic leukemia (T-ALL) and peripheral T cell lymphoma. A recent study showed that CD5-CAR-modified NK92 cells can kill a variety of T cell leukemia and lymphoma cell lines as well as primary tumor cells *in vitro* and in xenograft mouse models of T-ALL (Chen et al., 2017).

In addition to T-ALL, CD19-CAR-modified NK cell-based immunotherapy can be used to treat primary chronic lymphocytic leukemia (CLL) (Boissel et al., 2013), acute myeloid leukemia (AML, ClinicalTrials.gov.NCT00995137), myelodysplastic syndromes (Gleason et al., 2014), and B cell leukemia and lymphoma (Oelsner et al., 2017). The cytotoxicity of NK92 cells expressing CD20-CAR against primary CLL cells is superior to the cytotoxicity of NK92 cells expressing IgG Fcγ receptor III (FcγRIII, also known as CD16) combined with anti-CD20 monoclonal antibodies, such as rituximab or ofatumumab (Boissel et al., 2013).

Interestingly, trogocytosis can be used as a non-viral method to modify NK cells. Conventionally, immune cells can be directly modified using CAR viral particles. However, the authors used anti-CD19-CAR particles to transfect the K562 cell line (the first human immortalized myelogenous leukemia line with MHC class I deficiency). After mixing CD19-CAR-modified K562 cells with human primary NK cells isolated from PBMCs, CD19-CAR protein was transferred from CD19-CAR-modified K562 cells into NK cells via trogocytosis. The transferred CD19-CAR-modified NK cells functionally kill B cell acute lymphoblastic leukemia (B-ALL) cell lines and primary B-ALL cells derived from patients (Cho et al., 2014). This novel strategy could be a potential valuable therapeutic approach for modifying NK cells.

### CAR-modified NK cells to treat glioblastoma and neuroblastoma

It is well known that CAR-modified T cells face a unique set of challenges during the targeting of solid tumors (Gilham et al., 2012). The development of CAR-modified NK cells must overcome similar obstacles. Glioblastoma is one of the most lethal primary brain malignancies in adults and children, because of its highly invasive and metastatic characteristics (Magana-Maldonado et al., 2016). Neuroblastoma is a neuroendocrine tumor of early childhood and is the most common extracranial solid tumor that occurs in children (Matthay et al., 2016). It has been reported that NK92 cells have been developed to treat both glioblastoma and neuroblastoma *in vitro*. These cells have been modified to target neuroblastoma using a GD2 (disialoganglioside)-specific CAR (Esser et al., 2012) and to target glioblastoma using either an ErbB2 (origin in the *ERB-B* gene responsible for avian erythroblastosis virus)-CAR (Zhang et al., 2016) or an EGFR-CAR (Han et al., 2015). Therefore, it will be of interest to determine whether CAR-modified NK92 cells can treat both glioblastoma and neuroblastoma in clinical trials.

### CAR-modified NK cells to treat breast cancer

Breast cancer is the most common cancer of females in the U.S. (DeSantis et al., 2014). An adoptive cancer immunotherapy using CAR-modified NK92 cells has been rapidly developed. A stable NK92 cell line expressing an anti-human epidermal growth factor receptor 2 (HER2, also known as ErbB2)-CAR exhibited specific antitumor activity *in vivo* using an experimental non-obese diabetic (NOD) severe combined immunodeficiency (SCID) gamma (NSG) lung metastasis model (Schonfeld et al., 2015). Similarly, the combination of an EGFR-CAR-modified NK92 cell line therapy with the oncolytic herpes simplex virus 1 (oHSV-1) is a promising strategy for the treatment of EGFR-positive breast cancer that has metastasized to the brain (Chen et al., 2016).

### CAR-modified NK cells to treat multiple myeloma

Multiple myeloma (MM) is an incurable hematological malignancy that results from genetic mutations that occur during the process whereby B lymphocytes differentiate into plasma cells (Kyle and Rajkumar, 2008). Currently, a number of CAR-modified T cells have been developed for the treatment of MM (Liu et al., 2017a; Luetkens et al., 2017). However, few studies have reported using CAR-modified, primary NK cells for the treatment of MM. Current strategies using CAR-modified NK cell products to treat MM are focused on the CAR-modified NK92 cell line.

The cell surface glycoprotein CD2 subset 1 (CS1, also known as CRACC, SLAMF7, CD319, or 19A24) is a surface protein that is highly expressed on MM cells (Hsi et al., 2008; Tai et al., 2008). A previous study showed that CS1-CAR-modified NK92 cells inhibited MM tumor growth and prolonged survival of tumor-bearing NSG mice (Chu et al., 2014). CD138, also known as syndecan-1, is a primary diagnostic marker for MM (Akl et al., 2015). Therefore, CD138-CAR-modified NK92 cells have been used to treat MM in non-obese diabetic mice with severe combined immunodeficiency (Jiang et al., 2014).

### CAR-modified NK cells to treat prostate cancer metastases

Instead of using a conventional CAR containing the CD3zeta domain, a new CAR containing DNAX activation protein 12 (DAP12) has been proposed to modify NK cells (Topfer et al., 2015). Compared to CD3zeta, DAP12 is a signaling adaptor molecule involved in the signal transduction of stimulatory NK cell receptors, such as NKG2C (Lanier et al., 1998a), NKP44 (Campbell et al., 2004), KIR3DS1 (Carr et al., 2007), and KIR2DS1/2/5 (Lanier et al., 1998a, b; Smith et al., 1998; Della Chiesa et al., 2008; Hayley et al., 2011). Therefore, a DAP12-based, anti-prostate stem cell antigen CAR could be used to modify NK cells for the treatment of prostate cancer (Topfer et al., 2015). The data demonstrate that the use of NK cells modified with DAP12-based CARs is a promising approach for adoptive immunotherapy.

In conclusion, in addition to enhancing antibody-dependent cell-mediated cytotoxicity (ADCC) and blocking inhibitory receptor functions using KIR and other inhibitory receptors antibodies, modifying NK92 cell line to target various tumors has been a major, current effort in the field of NK cell-based immunotherapy.

## CAR-MODIFIED NK CELL-BASED IMMUNOTHERAPY TO TREAT INFECTIOUS DISEASES

Although great advances in human immunodeficiency virus (HIV, one of the major human pathogens) treatment have been made in most developed countries, the HIV/acquired immune deficiency syndrome (AIDS) pandemic continues to be a major public health problem in the majority of developing countries. In this section, we focus on the potential applications of CAR-modified NK cells for the treatment of HIV infections.

Eradication of HIV from infected individuals remains a major medical challenge (Siliciano, 2014; Churchill et al., 2016; Mzingwane and Tiemessen, 2017). Although combination antiretroviral therapy (cART) has dramatically reduced HIV-related morbidity and mortality (Palella et al., 2006), it fails to eliminate HIV *in vivo* due to the persistence of replication-competent proviruses in long-lived, latently infected cells, also known as HIV reservoirs. Recently, the use of CAR-modified CTLs to target cancer has become a promising approach for cancer immunotherapy and represents a broad-based approach by which T cells can be engineered to overcome antigen restriction or mutation (Wang and Wang, 2017). To test whether CAR-modified CTLs can be redirected toward HIV-infected target cells, several groups have developed different CAR-modified T cells for this purpose (Liu et al., 2015; Zhen et al., 2015; Ali et al., 2016; Liu et al., 2016), including one of earliest CAR-modified T cell clinical trials designed to treat HIV (Yang et al., 1997). Although the field is extensively investigating CAR-modified T cells to treat chronic HIV infections, the use of CAR-modified NK cell-based immunotherapy to treat chronic HIV infections has several advantages, as described below.

### NK cells can directly recognize HIV-infected target cells

The adaptive immune system, including T cells and B cells, plays an essential role in HIV control during the acute and chronic phases of infection (McMichael et al., 2010; Jones and Walker, 2016). However, an increasing body of evidence shows that NK cell dysfunction is closely associated with HIV disease progression (Alter and Altfeld, 2006; De Maria and Moretta, 2008; Iannello et al., 2008a, b; Alter and Altfeld, 2009; Fadda and Alter, 2011; Jost and Altfeld, 2012; Kramski et al., 2013; Hens et al., 2016; Scully and Alter, 2016).

HIV-infected target cells can upregulate the ligands recognized by NK cells (Richard et al., 2010). For example, HIV-infected primary NK cells upregulate unique long (UL) 16 (a human cytomegalovirus glycoprotein) binding protein (ULBP)-1 and -2, but not ULBP-3, MHC class I polypeptide-related sequence (MIC)-A, or MIC-B (Ward et al., 2009). ULBP-1 and -2 can strongly induce NKG2D -mediated NK cell immune responses in humans (Ogasawara and Lanier, 2005; Bryceson and Ljunggren, 2008; Le Bert and Gasser, 2014).

### NK cells can rapidly secret Interferon (IFN)-gamma to initiate a cellular anti-HIV response

The production of IFN-gamma by immune cells is an essential defense mechanism against many viral, bacterial, and parasitic infections (Borges da Silva et al., 2015). NK cells are a major innate source of IFN-gamma (Travar et al., 2016; Waggoner et al., 2016). Although CD4^+^ T cells are a major adaptive source of IFN-gamma, their ability to respond to infection is slower than that of NK cells. NK cells produce IFN-gamma rapidly during infections, which is essential to drive the differentiation of CD4^+^ T cells and induce adaptive immune responses. During HIV infections, NKG2A^+^ NK cells can quickly produce IFN-gamma to control HIV replication (Lisovsky et al., 2015), suggesting the important role of NK cells in the control of early HIV infection.

### NK cells can kill HIV-infected target cells via ADCC

ADCC is one of the most important functions of NK cells. ADCC plays an important role in controlling HIV infections (Chung et al., 2008). For example, ADCC activity was modestly protective in the RV144 HIV vaccine (an estimated efficacy of 31.2% at preventing HIV infection among South African adults) trial (Haynes et al., 2012). Enhancing ADCC activity could be a potential strategy for HIV vaccine development.

### NK cells can kill T follicular helper (Tfh) cells, a critical HIV reservoir

CD4^+^ PD-1^high^ Tfh cells are a newly identified virus reservoir in HIV-1 patients. The size of HIV-1 reservoirs positively correlates with the numbers of PD-1-expressing cells (Chomont et al., 2009; Hatano et al., 2013), as PD-1 expression marks cells that are more likely to harbor HIV-1 (Chomont et al., 2009; Deeks et al., 2012). Tfh cells in lymph nodes (LNs) are PD-1 positive and serve as the major CD4^+^ T cell compartment for HIV-1 infection, replication, and production (Perreau et al., 2013). Thus, Tfh cells in LNs and peripheral blood (PB) are also likely to be a key cellular reservoir for latent HIV-1 (Vinuesa, 2012; Pallikkuth et al., 2015). Although PD-1 expression is a hallmark of exhausted T cells during chronic infections (Wherry, 2011), PD-1^high^ Tfh cells do not experience exhaustion during chronic infections (Choi et al., 2013), indicating that they may have superior abilities to self-renew, resist apoptosis, and survive for extremely long periods of time during HIV-1 infection. Importantly, a recent study showed that NK cells can suppress CD4^+^ T cells and Tfh cells in a perforin-dependent manner during the first few days of infection (Rydyznski et al., 2015), resulting in a weaker germinal center (GC) response and diminished immune memory. One of current efforts in the search for an HIV cure includes disrupting GC formation, thereby reducing HIV reservoirs.

### NK cell KIR is associated with HIV selection pressure

Effective NK cell responses impact HIV-1 progression (Fauci et al., 2005; Alter and Altfeld, 2006; Alter and Altfeld, 2009; Alter and Altfeld, 2011; Alter et al., 2011; Altfeld et al., 2011; Funke et al., 2011; Jost and Altfeld, 2012, 2013; Altfeld and Gale, 2015). Killer cell immunoglobulin like receptor, three Ig domains and short cytoplasmic tail 1 (KIR3DS1), in particular, appears to inhibit HIV-1 replication *in vitro* (Martin et al., 2002; Alter et al., 2007; Jost and Altfeld, 2013) and be associated with slower AIDS progression in HIV-1-infected patients (Martin et al., 2002; Pascal et al., 2007; Long et al., 2008). Interestingly, KIR3DS1^+^ NK cells express high levels of IFN-γ (indicating enhanced cytokine secretion) and CD107a (indicating enhanced degranulation and cytotoxicity) in adults who were recently infected with HIV-1 (Long et al., 2008). In summary, the combination of KIR on NK cells and host human leukocyte antigen (HLA) can affect HIV progression.

### NK cells cannot be effectively infected by HIV

HIV primarily infects CD4^+^ T cells. Whether NK cells can be infected by HIV is controversial (Funke et al., 2011), although the consensus in the field is that HIV cannot effectively infect the majority of NK cells, because NK cells present in peripheral blood lack CD4 expression, and no proviral DNA can be detected in NK cells from HIV patients (Mavilio et al., 2003; Altfeld et al., 2011). A subpopulation of NK cells (<7%) was found to be sensitive to infection by HIV-1 *in vitro* (Chehimi et al., 1991; Scott-Algara et al., 1993; Valentin et al., 2002; Bernstein et al., 2009), but these data must be verified *in vivo*. In general, the majority of NK cells cannot be infected by HIV naturally, which makes NK cells attractive effector cells for the treatment of HIV. Importantly, NK cell lines that are currently used in clinical trials are CD4 negative, which means that these NK cell lines cannot be infected by HIV.

### CAR-modified NK cell immunotherapy for HIV

Despite the increasing body of evidence showing NK cell involvement in the control of HIV infection, HIV can affect NK cell phenotype and function during HIV infection, including the cytokine/chemokine production, activation, and cytotoxicity of NK cell subsets. Therefore, genetically modified NK cells, designed to enhance innate immunity, are essential for the development of a novel strategy to control infectious diseases, especially HIV. An early human NK3.3 reporter cell line can be genetically modified to express CD4zeta using a retroviral transduction approach. These CD4zeta-expressing NK cells can specifically kill NK-resistant tumor cells expressing the relevant ligand, HIV envelope glycoprotein 120 (gp120), or CD4^+^ T cells infected with HIV (Tran et al., 1995). Human NK3.3 cells can be readily activated via CD4zeta-based CARs to target both tumor and virus-infected cells, demonstrating early evidence that CAR-modified NK cells have the potential to be used to treat HIV infection.

Interestingly, a recent study reported that CAR-modified hematopoietic stem/progenitor cells (HSPCs) could differentiate into functional NK cells in humanized mice (Zhen et al., 2015). These NK cells are resistant to HIV infection and suppress HIV replication *in vivo*. The significance of this study is that CAR-modified HSPCs can differentiate into functional NK cells. This strategy provides a new approach to generate NK cells for immunotherapy. Manipulation of a patient’s immune cells usually presents many challenges. For example, the first problem is that there are insufficient numbers of NK cells that can be obtained from patient’s peripheral blood. The second problem is that NK cells that are freshly isolated from patients usually do not exhibit normal functions, compared with NK cells from healthy individuals. The hematopoietic development of CD4zeta-CAR-expressing NK cells from genetically modified HSPCs can provide a stable, functional, innate immune response to HIV, as a rapid, early immune response is important to control HIV replication. Additionally, CAR-modified HSPCs may possess the ability to produce NK cells over time, since one of the challenges of NK cell-mediated immunotherapy is the short life span of NK cells that are isolated from a patient’s peripheral blood.

In summary, in the field of HIV research, extensive studies have been focused on the roles of T and B cells in HIV infection. An increasing body of evidence indicates the importance of NK cells in HIV infection and HIV disease progression.

## THE CAR-MODIFIED NK CELL IS

Adoptive cell-based therapy using CAR-modified NK cells has the potential to extend the survival of cancer patients by enhancing the antitumor effectiveness of CAR-modified cells. In this context, an important question is emerging: How does one efficiently and rapidly choose the best CAR-modified cells for cancer patients? Specifically, researchers from different laboratories are generating different CARs with minor modifications. However, before these CAR-modified cells can enter clinical trials, it is essential that they can be evaluated precisely for their quality and efficacy in a cost-effective manner. Additionally, the speed at which these CAR T cells can be clinically tested is limited by the current, time-consuming, costly, and labor-intensive conventional approaches used to evaluate efficacy. In the field of basic immunology, T cell efficacy is not only controlled by the specificity and avidity of the tumor antigen and T cell interaction, but it also depends on a collective process, involving multiple adhesion and regulatory molecules, spatially organized at the T cell IS.

Here, we review the current progress in CAR-modified T cell and NK cell ISs, with a focus on CAR-NK ISs. The main message in this section is that the NK IS is critical to the understanding of the fundamental mechanisms underlying the cytotoxicity and side effects of CAR-modified T cell- or NK cell-based immunotherapy. Specifically, generation and modification of novel CAR-modified immune cells to target solid cancers and other diseases has been a major effort in the field of immunotherapy. The time has come for scientists to understand the fundamental mechanisms of CAR immunobiology. Lack of such knowledge is an important issue because, without it, choosing the best CAR-modified immune cells for patients with solid tumors or other diseases is highly unlikely.

### The background of IS

The NK cell IS (Davis et al., 1999) was first described between peripheral blood NK cells in the YTS cell line and various transfectants of 721.221 (a B cell line derived by mutagenesis that does not express MHC class I molecules (Shimizu et al., 1988)). The cell biology of NK cells and their IS has been reviewed by others (Bromley et al., 2001; Davis, 2002; Lagrue et al., 2013). The original concept of NK IS is derived from T cell IS. Control of T cell activation and modulation of T cell function not only depend on the TCR-epitope-MHC complex interaction, but also on a collective process that involves multiple adhesion and regulatory molecules spatially organized at the T cell-APC interface, forming the T cell IS (Fooksman et al., 2010). Fixed-cell imaging studies on T-cell APC conjugates (Monks et al., 1998) and multiple dynamic studies with planar bilayers (Grakoui et al., 1999; Campi et al., 2005; Varma et al., 2006) have illuminated the molecular organization of physiological T cell activation. The IS corresponds to a concentric structure of discrete domains with TCRs and CD3 molecules occupying the central region (central supramolecular activation cluster; cSMAC), which is surrounded by an outer ring of adhesion molecules in the peripheral SMAC (pSMAC). Besides this classic model largely studied with naïve or resting CD4^+^ T cells, it has become clear that there are several forms of IS. Effector CD8^+^ CTL and NK cells can form both cytotoxic IS (leading to killing), stimulatory IS (leading to cytokine secretion), and inhibitory IS (Stinchcombe and Griffiths, 2003; Liu et al., 2009; Liu et al., 2012; Jang et al., 2015). The structure of NK IS appears less organized than T cell IS. For example, the distribution of major IS components, including activation cluster (e.g., CD16) and integrin molecules (e.g., LFA-1), at NK IS formed on the glass-supported planar lipid bilayer containing human IgG1 Fc portion (a ligand for CD16) and ICAM-1 (a ligand for LFA-1) is not well organized (Liu et al., 2009). The effector CTL IS contains a distinct central secretory domain (Stinchcombe et al., 2006), with granule secretion controlled by centrosome delivery to the plasma membrane. Integrity of the pSMAC ring is also important for effective killing (Anikeeva et al., 2005). In summary, basic cytotoxic IS (including CTL and NK cell) structure and molecule pattern can vary, but the function of directed secretion at cytotoxic IS is similar, which leads to killing target cells through the polarized release of lytic granules at IS.

### Current approaches for studying IS

**Figure 1:**
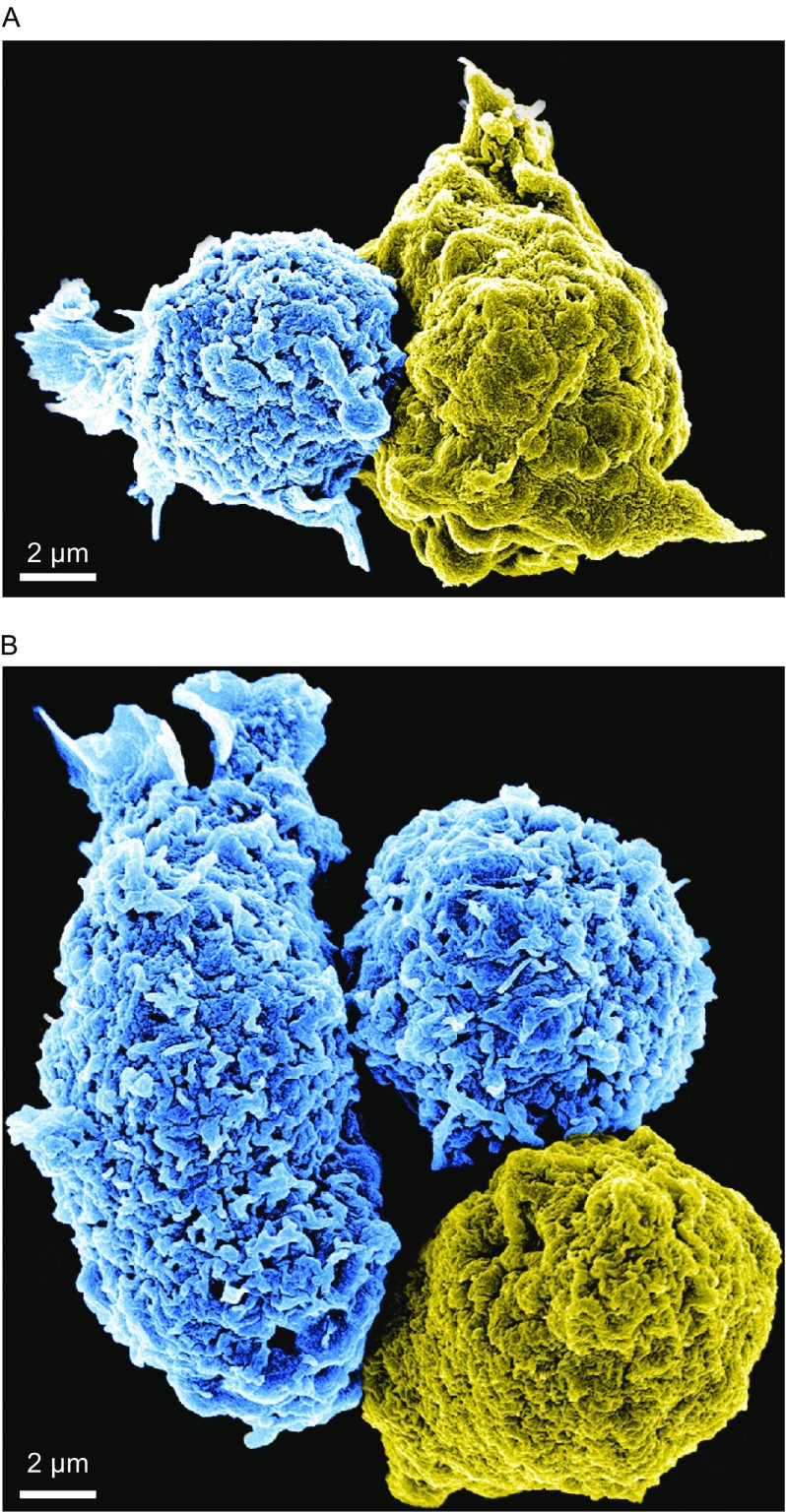
The CAR-modified NK cell IS. (A) One CAR-modified NK92 cell (blue) interacts with a tumor cell (yellow) through an IS. (B) Two CAR-modified NK92 cells (blue) interact with a tumor cell (yellow) through two different ISs. These are two representative scanning electron micrographs using two different colors

**Figure 2:**
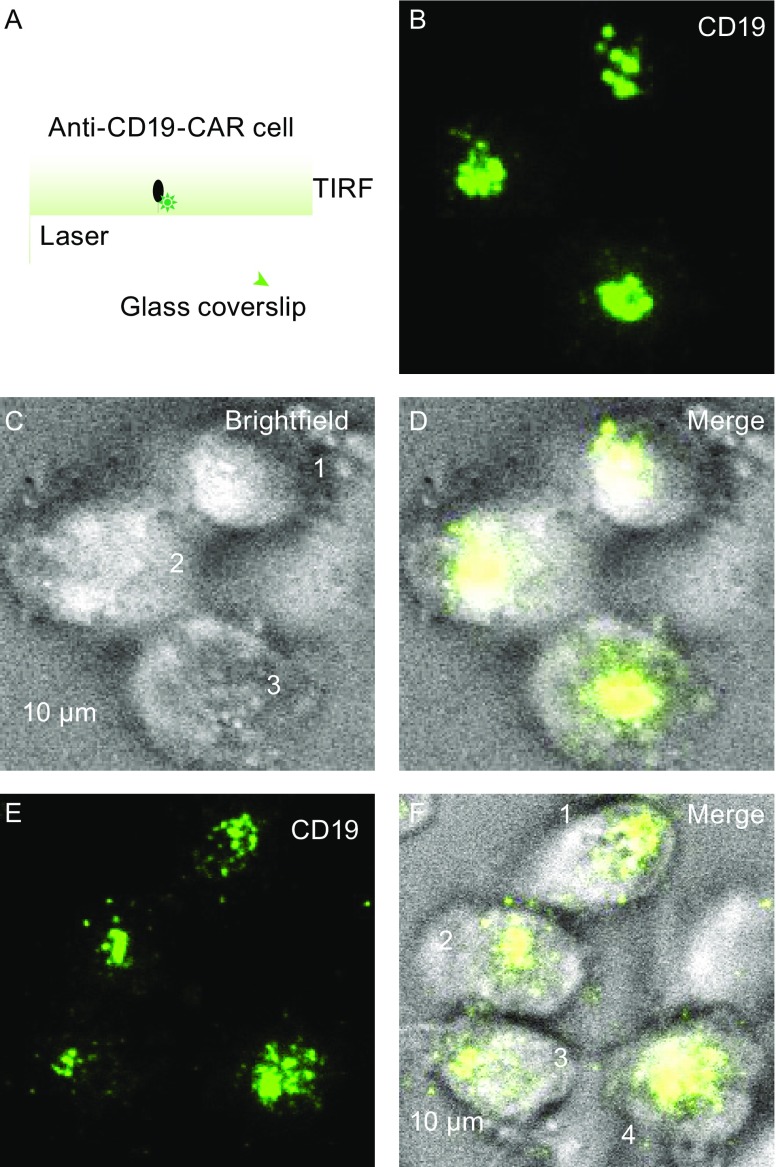
**CAR-modified NK cell ISs on a glass-supported, planar lipid bilayer.** (A) Schematic depiction of a TIRF setup in which a lipid bilayer contains a fluorescence dye-labeled tumor antigen (green). TIRF (B), brightfield (C), and merge (D) images of CAR-modified NK cell IS formation on a glass-supported, planar lipid bilayer carrying an Alexa488-labled human CD19 protein (green). The three CAR-modified NK cells that contacted the lipid bilayer, as determined by the central accumulation of tumor antigen under TIRF microscopy, are numbered. Representative TIRF (E) and merge (F) images of CAR-modified NK cells are shown. Four individual CAR-modified NK cells, fixed at 30 min after addition to the bilayer carrying CD19-Alexa Fluor 488, are numbered. The images are representative of at least 100 cells from three independent experiments

Conventional fluorescence microscopy of immune cells represents the most common imaging strategy to investigate the IS (Jang et al., 2015; Zheng et al., 2015a). A high-resolution imaging approach, including electron microscopy (Fig. 1) and fluorescence microscopy in combination with the glass-supported planar lipid bilayer system (Fig. 2), can provide a new look at the structure of the CAR-modified cell IS, allowing a determination of the precise relationship between IS quality and the effectiveness of CAR-modified cells. Unlike Western blot (WB) and immunoprecipitation (IP), which only assess the average signaling state, microscopy-based assays, including the advent of commercially available high-resolution optical microscopes, such as total internal reflection fluorescence (TIRF) microscopy (Liu et al., 2009; Liu et al., 2012) and stimulated emission depletion (STED) microscopy (Zheng et al., 2015a; Zheng et al., 2015b), reveal structure, function, and signaling (i.e., quality) of IS. Also, the IS is one of the most pivotal communication strategies used by immune cells (Jang et al., 2015). In addition to the cell-cell conjugation system, the structure, function, and signaling cascades at the IS have been further confirmed by imaging T cell interactions with a glass-supported, planar lipid bilayer, which contains the MHC-peptide complex and other costimulatory molecules (Tozeren et al., 1992; Grakoui et al., 1999; Lee et al., 2002, 2003; Mossman et al., 2005). The general consensus in the field of immunology is that a glass-supported, planar lipid bilayer system can mimic target cells for the study of the IS at high resolution (Dustin et al., 2007; Choudhuri et al., 2014; Zheng et al., 2015a; Bertolet and Liu, 2016). Previous studies have demonstrated that IS quality determines the efficacy of T cell-mediated cytotoxicity (Grakoui et al., 1999; Jenkins et al., 2009; Dustin and Long, 2010). Similarly, an obvious question is whether CAR-modified T and NK cells can form functional ISs. Indeed, CAR-modified NK cells can form functional ISs on a glass-supported, planar lipid bilayer (Fig. 2), in which the images of fixed CAR-modified NK cells on lipid bilayers reveal the central accumulation of CD19, which is reminiscent of the central cluster of TCRs and B cell receptors at the synapse (Fooksman et al., 2010; Harwood and Batista, 2010). Thus, the glass-supported planar lipid bilayer system can serve as a reductionist approach to study CAR IS. Additionally, CAR T and NK cells do form ISs on the glass-supported lipid bilayer system.

### The rationale for the investigation of CAR IS

A critical factor affecting the efficacy of NK cells for CAR-modified NK cell-based immunotherapy is the tumor microenvironment, which is a key element in the exploitation of NK cells for the treatment of solid tumors (Gras Navarro et al., 2015). The infiltration of NK cells into solid tumors has been reported previously (Burke et al., 2010; Pietra et al., 2016). The status of tumor-infiltrating NK cells correlates with the prognosis for melanoma (Burke et al., 2010). The interactions between NK cells and the tumor microenvironment include: (1) soluble factor production (transforming growth factor-beta, indoleamine 2,3-dioxygenase, interleukin (IL)-4, prostaglandin E2) by tumor cells or other cells in the tumor microenvironment (Castriconi et al., 2003; Marcenaro et al., 2005); (2) inhibitory cells at the tumor site, which include myeloid-derived suppressor cells, regulatory T cells, tumor-associated macrophages, tumor-associated fibroblasts, and tumor cells (Pietra et al., 2016); and (3) dysfunctional NK cells at the tumor site. These dysfunctional NK cells are characterized by the upregulated expression of several inhibitory receptors, such as PD-1 (Keir et al., 2008; Benson et al., 2010), and the downregulation of critical, stimulatory NK receptors, such as NKG2D (Krockenberger et al., 2008). Among these factors, one of the most important cellular interactions in the tumor microenvironment is the interaction between NK cells and tumor cells, also known as the NK IS (Davis et al., 1999; Davis, 2002; Orange, 2008). A CAR-modified T cell or NK cell must form an effective IS with susceptible target cells to kill them.

Our current knowledge lacks a complete understanding of CAR biology and an effective, unanimously recognized approach to predict the effectiveness of CAR-modified cells. Using the IS quality to predict the efficacy of immunotherapy and side effects of CAR T/NK cells will introduce a superior tool or parameter into the field of immunotherapy. Specifically, to assess the effectiveness of CAR-modified cells, the quality of the IS formed by these cells and susceptible target cells, including virus-infected cells and tumor cells, as well as the glass-supported planar lipid bilayer system for mimicking the surface of an tumor cell or infected target cell, can be quantified.

Traditional biochemical and cell biological approaches for the analysis of signaling pathways (e.g., WB and IP) rely on homogenized cellular extracts from millions of cells. While fast and inexpensive, these methods do not reveal critical spatiotemporal parameters or the dynamics of intracellular signal transduction within the IS.

Although tremendous progress has been made in the basic research of the IS, to date, no study has addressed how the IS controls CAR-modified cell function. Such knowledge is important for optimally choosing the best CAR-modified T cells for cancer patients, as well as a tool to evaluate the efficacy of CAR T/NK cells. Currently available strategies to evaluate the effectiveness of CAR T cells include conventional *in vitro* methods, such as cytokine secretion, cytotoxicity, proliferation, ratio of CD4/CD8 cells, and long-term killing assays, as well as *in vivo* mouse models. To assess the homing, persistence, and antitumor activity of CAR T cells *in vivo*, scientists use a SCID mouse model and the *in vivo* imaging system (Bhaumik and Gambhir, 2002; Kim et al., 2004; Vera et al., 2006; Wang et al., 2009; Morse and Tannous, 2012). However, currently available *in vitro* and *in vivo* analyses are time consuming, costly, and labor intensive; thus, a new approach is urgently needed to solve this problem (Geldres et al., 2016). Accordingly, more sophisticated, high-resolution techniques are warranted to quantitate the function of CAR-modified cells, as described above.

In summary, CAR-modified T cell- and NK cell-mediated ISs are essential to the understanding of the efficacy of CAR-modified T cells and NK cells and their toxicities.

## PERSPECTIVES

Recent progress in the understanding of NK cell biology and immunology, including memory NK cells (Adams et al., 2016; Fehniger and Cooper, 2016) and the signaling pathways of immune receptors on NK cells (Long et al., 2013), has established the foundation for harnessing the power of NK cells for innovative immunotherapies. However, there are many potential challenges regarding the use of NK cell-based immunotherapy in the future, as detailed below.

### NK cell rapid expansion, cryopreservation, and shipping

Rapid NK cell expansion technique is urgently needed. Recent studies using 4-1BB (also known as CD137) ligand (4-1BBL/CD137L) and IL-21 expressing K562 cells as feeder cells can be used to rapidly expand NK cells *in vitro* (Denman et al., 2012). However, the characterization and application of these cells for the treatment of patients is essential to ensure that the cells are functional and healthy. In addition, specific NK cell expansion is also needed to advance NK cell immunotherapy *in vivo*. One potential issue regarding NK cell expansion *in vitro* using irradiated feeder cells in the presence of cytokine IL-2 is that naïve immune cells become exhausted or senescent after rapid proliferation and differentiation (Keir et al., 2008). Indeed, CAR-modified immune cells express exhaustion markers such as PD-1 (John et al., 2013; Cherkassky et al., 2016; Chong et al., 2016; Gargett et al., 2016). To solve the problem of immune cell exhaustion, one approach is to block PD-1 signaling in CAR-modified T cells (Cherkassky et al., 2016). Another potential strategy is to alter the metabolic pathway in CAR-modified T cells (Ping et al., 2017) or reinforce lymphocyte metabolism (Lim and June, 2017), given the existence of essential metabolic signaling in T cells (Buck et al., 2015). Therefore, it will be of interest to determine whether the alternation of metabolic pathways can enhance NK cell expansion without exhaustion.

At present, the expansion of CAR-modified T and NK cells requires *in vitro* stimulation of genetically modified T and NK cells using antibodies and cytokines. These antibody and cytokine-driven activation and expansion may negatively alter CAR-T/ NK cell functions. For example, CAR-modified immune cell exhaustion can be induced by the end of extensive expansion program, which is evident by the up-regulation of PD-1, TIM-3, and LAG-3 in CAR T cells (Long et al., 2015). Therefore, new modification and expansion strategies without induction of exhaustion may be developed *in vivo*, given immune cell exhaustion is a major factor for compromised immune responses against tumor and virus during chronic antigen stimulation (Virgin et al., 2009; Wherry, 2011). Additionally, the current expansion of CAR-modified immune cells for clinical applications takes at least 2–3 weeks, which becomes a significant hurdle for some patients. The “sleeping beauty transposon” or piggBac system, which is capable of delivering large (9.1–14.3 kb) transposable elements without a significant reduction in T cell efficacy (Maiti et al., 2013; Guerrero et al., 2014; Singh et al., 2014), in combination with genetically engineered artificial cells expressing membrane-bound IL-15 and 4-1BB ligands, has already been used for CAR-modified T cell immunotherapy. This approach may promise the rapid expansion of NK cells in the future.

After successful expansion, cryopreservation and transportation of NK cells are also essential to advance NK cell utility in the clinic. After cryopreservation (the freeze/thaw cycle) and transportation, recovered NK cells often experience a decrease in function. The viability of recovered NK cells can also be a potential issue for NK cell-based immunotherapy. Therefore, the development of new cryopreservation and transportation methods is urgently needed.

### Sources of NK cells

There are two sources of NK cells (autologous and allogeneic NK cells), which can be obtained from PBMCs, apheresis products, bone marrow, cord blood cells, embryonic stem cells, and induced pluripotent stem cells. Compared to primary NK cells isolated from blood and other sources, human NK92 cell line is easier and affordable. The clinical application of irradiated CAR-NK92 is safe and effective, as the use of NK cell lines can significantly reduce the cost of immunotherapy. Additionally, NK cells directly isolated from immunocompromised cancer patients usually have poor cytotoxicity and functionality, precluding their use. The future development of CAR-modified NK92 products promises to be both feasible and inexpensive.

### Strategies for long-lived, expandable NK cells

The life span of NK cells is generally shorter than that of CTLs. Increasing the life span of *ex vivo*-expanded NK cells has become a pivotal issue in immunotherapy. The advantage of the short life span of CAR-modified NK cells is that they have fewer off-target effects than CAR-modified T cells. Scientists are currently seeking a technique that will expand NK cells in a shorter time period for urgent clinical needs. K562-mbIL21-41BBL cells have recently been used to expand NK cells rapidly (Denman et al., 2012). In the future, it will be essential to produce both long-lived and quickly expandable NK cells, such as memory NK cells (Sun et al., 2011; Adams et al., 2016; Fehniger and Cooper, 2016), for generating CAR-modified NK products.

### Potential toxicity of CAR-modified NK cells and manufacturing costs

Generally, it is thought that NK cell-based immunotherapy results in less severe side effects than genetically modified T cell-based immunotherapy. However, a direct comparison of side effects between CAR-modified T cells and NK cells is not available. The routine management of CAR-modified NK cell toxicity is desirable. For example, the use of inducible caspase-9 in the construct (Di Stasi et al., 2011; Sadelain, 2011), causing the dimerization of caspase-9 by FK560, will induce CAR-modified NK cell apoptosis. This strategy limits the potential side effects of CAR-modified NK cells and minimizes other cellular damage, such as that from a cytokine storm. Similarly, the engineered synthetic Notchs (synNotchs) system in CAR-modified T cells (Roybal et al., 2016) may be applicable to CAR-modified NK cells. Furthermore, the current cost of CAR-mediated immunotherapy is high. An FDA-approved, CAR-mediated immunotherapy must undergo multi-phase clinical trials. The approximate costs for phase I, II, and III clinical trials are $2–5, $5–15, and $10–50 million, respectively. Meanwhile, the manufacturing of CAR-modified immune cells is costly, labor intensive, and time consuming. In the future, a closed, automated workflow system is essential to reduce the cost of generating CAR-modified T cells and NK cells.

### Enhancement of transfection efficiency for peripheral blood NK cells

One of the biggest obstacles to the use of gene-modified NK cells for immunotherapy has been the absence of an efficient gene transfer technique. Several technologies, including retroviral and lentiviral systems, have been used to enhance the transduction efficiency of NK cell lines and activated, primary NK cells (Lapteva et al., 2016). The subsequent challenge is to apply these approaches to resting NK cells isolated directly from peripheral blood that maintain their cellular functions.

### Development of “off-the-shelf” NK cell products

Development of “off-the-shelf” NK cell products is still in the concept stage. There are no universal NK cell products that can be used to treat a variety of tumors. The CAR-modified NK92 cell line may serve as a future “off-the shelf” NK product.

In conclusion, CAR-modified NK cells with manageable toxicities have emerged as a powerful, effective tool for fighting cancer and infectious diseases. To harness the power of these cells, basic research of the cell biology and immunology of CAR-modified NK cells, with a focus on the CAR-modified NK cell-mediated *in vitro* and *in vivo* IS, is essential.
