# Erratum to: Chimeric antigen receptor (CAR)-modified natural killer cell-based immunotherapy and immunological synapse formation in cancer and HIV

**DOI:** 10.1007/s13238-017-0427-1

**Published:** 2017-06-23

**Authors:** Dongfang Liu, Shuo Tian, Kai Zhang, Wei Xiong, Ndongala Michel Lubaki, Zhiying Chen, Weidong Han

**Affiliations:** 1 Center for Inflammation and Epigenetics, Houston Methodist Research Institute, Houston, TX 77030 USA; 2 Department of Microbiology and Immunology, Weill Cornell Medical CollegeCornell University, New York, NY 10065 USA; 3 Institute of Basic Medicine, College of Life Sciences, Chinese PLA General Hospital, Beijing, 100853 China

## Erratum to: Protein Cell DOI 10.1007/s13238-017-0415-5

In the original publication of this article Fig. 2A is incorrectly published. The correct version of Fig. 2A and the following corrections are provided in this erratum.

**Figure 2:**
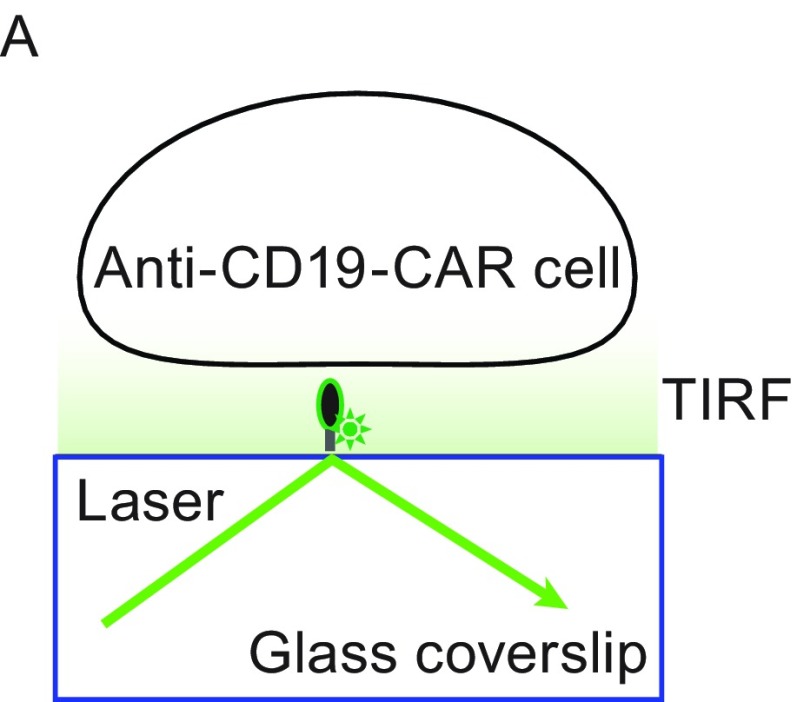
**CAR-modified NK cell ISs on a glass-supported, planar lipid bilayer**. (A) Schematic depiction of a TIRF setup in which a lipid bilayer contains a fluorescence dye-labeled tumor antigen (green). TIRF (B), brightfield (C), and merge (D) images of CAR-modified NK cell IS formation on a glass-supported, planar lipid bilayer carrying an Alexa488-labled human CD19 protein (green). The three CAR-modified NK cells that contacted the lipid bilayer, as determined by the central accumulation of tumor antigen under TIRF microscopy, are numbered. Representative TIRF (E) and merge (F) images of CAR-modified NK cells are shown. Four individual CAR-modified NK cells, fixed at 30 min after addition to the bilayer carrying CD19-Alexa Fluor 488, are numbered. The images are representative of at least 100 cells from three independent experiments

Under the section “The rationale for the investigation of CAR IS” the line starting with “the surface of an tumour cell or infected target cell” is incorrect, it should be read as “the surface of a tumour cell or infected target cell”.

Under the section of “COMPLIANCE WITH ETHICS GUIDELINES” the line starting with “This article does not contain any studies” is incorrect, it should be read as “This article does not contain any studies with human or animal subjects performed by any of the authors”.

Under the section “Development of “off-the-shelf” NK cell products” the line starting with “The CAR-modified NK92 cell line may” is incorrect, it should be read as “The CAR-modified NK92 cell line may serve as a future “off-the-shelf”.

Under the section “The background of IS” the line starting with “between peripheral blood NK cells in the YTS cell line and various transfectants” is incorrect, it should be read as “between peripheral blood NK cells and various transfectants”.

